# Medical education in pharmacogenomics—results from a survey on pharmacogenetic knowledge in healthcare professionals within the European pharmacogenomics clinical implementation project Ubiquitous Pharmacogenomics (U-PGx)

**DOI:** 10.1007/s00228-017-2292-5

**Published:** 2017-07-02

**Authors:** Katja Susanne Just, Michael Steffens, Jesse Joachim Swen, George P. Patrinos, Henk-Jan Guchelaar, Julia Carolin Stingl

**Affiliations:** 1 Research Division, Federal Institute for Drugs and Medical Devices, University Bonn Medical School, Kurt-Georg-Kiesinger-Allee 3, 53175 Bonn, Germany; 2 Department of Clinical Pharmacy & Toxicology, Leiden University Medical Center, Leiden, The Netherlands; 3 Department of Pharmacy, School of Health Sciences, University of Patras, Patras, Greece; 4 University Bonn, Medical Faculty, Centre for Translational Medicine, Bonn, Germany

**Keywords:** Pharmacogenomics, Pharmacogenetics, Clinical pharmacology, Cultural diversity, Medical education

## Abstract

**Purpose:**

Due to the diversity within Europe, the implementation of pharmacogenomic testing in clinical practice faces specific challenges. In the context of the European pharmacogenomics implementation project “Ubiquitous Pharmacogenomics” (U-PGx; funded by the European Commission), we studied the current educational background.

**Methods:**

We developed a questionnaire including 29 questions. It was spread out to healthcare professionals working at the future implementation sites (in Austria, Greece, Italy, Netherlands, Slovenia, Spain and Great Britain) of the U-PGx project in preparation of an educational programme. Aim of the survey was to analyse the current educational situation at the implementation sites.

**Results:**

In total, 70 healthcare professionals participated in the survey. Of participants, 84.3% found pharmacogenomics relevant to their current practice, but experience was still rare. More than two-thirds (65.7%) did not order nor recommend a pharmacogenomic test in the past year. This was mainly attributed to not having enough knowledge on pharmacogenomics (40.0%). Needs were identified in application of pharmacogenomics (identifying drugs 41.4%, interpreting test results 37.2%) as well as in underlining mechanisms (better knowledge on drug metabolism 67.1%, better knowledge on basic principles of pharmacogenomics 60.0%).

**Conclusions:**

This study analysed the specific attitudes, experience and education on pharmacogenomics of future users. There was a general positive attitude and interest towards pharmacogenomic testing. However, the grade of own experience, and knowledge about application and interpretation of pharmacogenomics caused uncertainty. Thus, education and training programmes may be helpful for implementation of pharmacogenomics at a homogenous level within Europe.

**Electronic supplementary material:**

The online version of this article (doi:10.1007/s00228-017-2292-5) contains supplementary material, which is available to authorized users.

## Introduction

In the context of the study, Ubiquitous Pharmacogenomics, pharmacogenomic testing was understood mainly for assessment of the variability of genes affecting drug disposition, metabolism and drug transport leading to individual responses to drugs [1]. By genotyping, testing for established gene variants, it is assumed to improve drug efficacy and safety [2, 3]. Pre-emptive, prospective, genotyping to make individualised drug therapy feasible is seen to contribute to personalised medicine [4]. Pre-emptive genotyping is thereby thought to be used to decide on the right drug for the right patient as well as the right dose [5]. Potential benefits of pharmacogenomics (PGx) have been defined such as predicting intended response to medication by more accurate dosing, avoiding adverse drug reactions and therefore enhancing drug safety and reducing health care costs [6]. However, in the field of PGx, it is challenging to collect sufficient sample sizes for analysing effectiveness in clinical trials owing the rarity of PGx variants and population heterogeneity [2, 7].

In the USA, there are medical centres with large current programmes implementing pharmacogenomics into clinic using pre-emptive testing facing specific challenges [5]. The need of standardised education programmes on pharmacogenomics for pharmacists and physicians was addressed [8, 9]. Concordantly, some Pharmacogenomics Educational Programs were established [5, 10–14].

In Europe, the uptake of PGx into clinical care seems to fall behind [15]. Challenges in establishing pharmacogenomics into clinic in Europe have been identified such as cost-effective studies with a need for data and tool sharing between countries, training programmes and education of medical staff [16–18] also addressing the diversity of health care systems within Europe. The availability of adequate lab tests is a crucial step in increasing the uptake of pharmacogenomics into clinic [19, 20]. Different backgrounds and specialities in health care might show varying affinity to pharmacogenomic diagnostic [9, 21]. Furthermore, different payment and funding structures compared to the USA are a challenge for clinical utility of PGx in Europe and within different European countries [16, 22]. In this context, one has to see the importance of a harmonised educational environment in pharmacogenomics and its clinical application in Europe, as knowledge and education have been defined several times as crucial step for successful implementation [5, 8–10, 12, 16, 23–25].

The Ubiquitous Pharmacogenomics (U-PGx) project which was funded by the European Commission is aiming to implement pre-emptive genotyping in seven existing European health care environments (implementation sites) [1, 23, 26]. In this context, education of participating health care professionals and further is in the focus of the project. However, diversity of health care systems might be a challenging factor. We intended to study the current knowledge about pharmacogenomics and the attitudes of clinicians towards pharmacogenomic testing at seven implementation sites in European countries in which the project will be run.

### Materials

We conducted an internet-based survey on knowledge and attitude towards PGx among 70 health care professionals related to the U-PGx implementation project. This survey was voluntary and participants were informed about anonymity and potential use of the results for publication. Aim of the survey was to analyse the current situation at implementation sites involved in the U-PGx project. Furthermore, results of the survey should act as a basis for development of an educational programme enabling PGx testing in clinic. Therefore, a questionnaire with 29 questions was designed. The questionnaire was composed in a stepwise procedure, including internal and external review steps. Some questions were included on basis of a search of literature to make the questionnaire comparable to others. When a first draft of the questionnaire was ready, it was sent to the seven implementation site leaders for feedback, and their comments were subsequently included. The final questionnaire was approved by all clinical sites.

The questionnaire was piloted in a cohort of clinicians in Germany (final questionnaire is provided in the supplementary material).

The survey was designed using SurveyGizmo© and was made accessible via the official U-PGx website in a password-protected manner. Since pharmacogenomics testing will be implemented in different clinical sites in the context of the U-PGx study, this survey was performed prior to the start of the implementation study involving clinicians at the clinical sites working in direct patient contact and with those, who are deciding about drug treatment and therapy adjustment. As the number of persons who were reached by the pharmacogenomics testing service varied at the different sites, the number of clinicians from each country to be contacted was different. The implementation sites were in Austria, Greece, Italy, Netherlands, Slovenia, Spain, and the UK. The survey was online for two and a half months (from June 13 to August 31 2016). Analyses were conducted using the SurveyGizmo© report. Where necessary, this analysis was supplemented with analysis conducted in excel.

## Results

In total, 70 surveys were answered completely (responder rate 27.5%), from seven implementation sites involved in U-PGx. The implementation sites were very different in size, organisational structure, and medical orientation. Characteristics and numbers of participants in the survey are seen in Table 1.

**Table 1:** Characteristics of the healthcare professionals participating in the U-PGx survey (*n* = 70, involved in implementation project)

Gender	Female	52.9% (37)
	Male	47.1% (33)
Age (years)	Mean (range)	39 (25–67)
profession	Physician	75.7% (53)
	Pharmacist	15.7% (11)
	Other^a^	8.6% (6)
Primary practice setting	Hospital inpatient	50.0% (35)
	Outpatient	31.4% (22)
	Academia/research	17.1% (12)
	Other^b^	1.4% (1)
Work experience (years)	1	11.4% (8)
	2–5	21.4% (15)
	6–10	15.7% (11)
	11–20	30.0% (21)
	>20	21.4% (15)
Country	Austria	18.6% (13)
	Great Britain	20.0% (14)
	Greece	18.6% (13)
	Italy	12.9% (9)
	Netherlands	12.9% (9)
	Slovenia	1.4% (1)
	Spain	15.7% (11)

Data given in percentage (absolute number), except age (given as mean (range))

^a^Includes lab personal

^b^Not distinguishable

### Experience and attitude

**Fig. 1:**
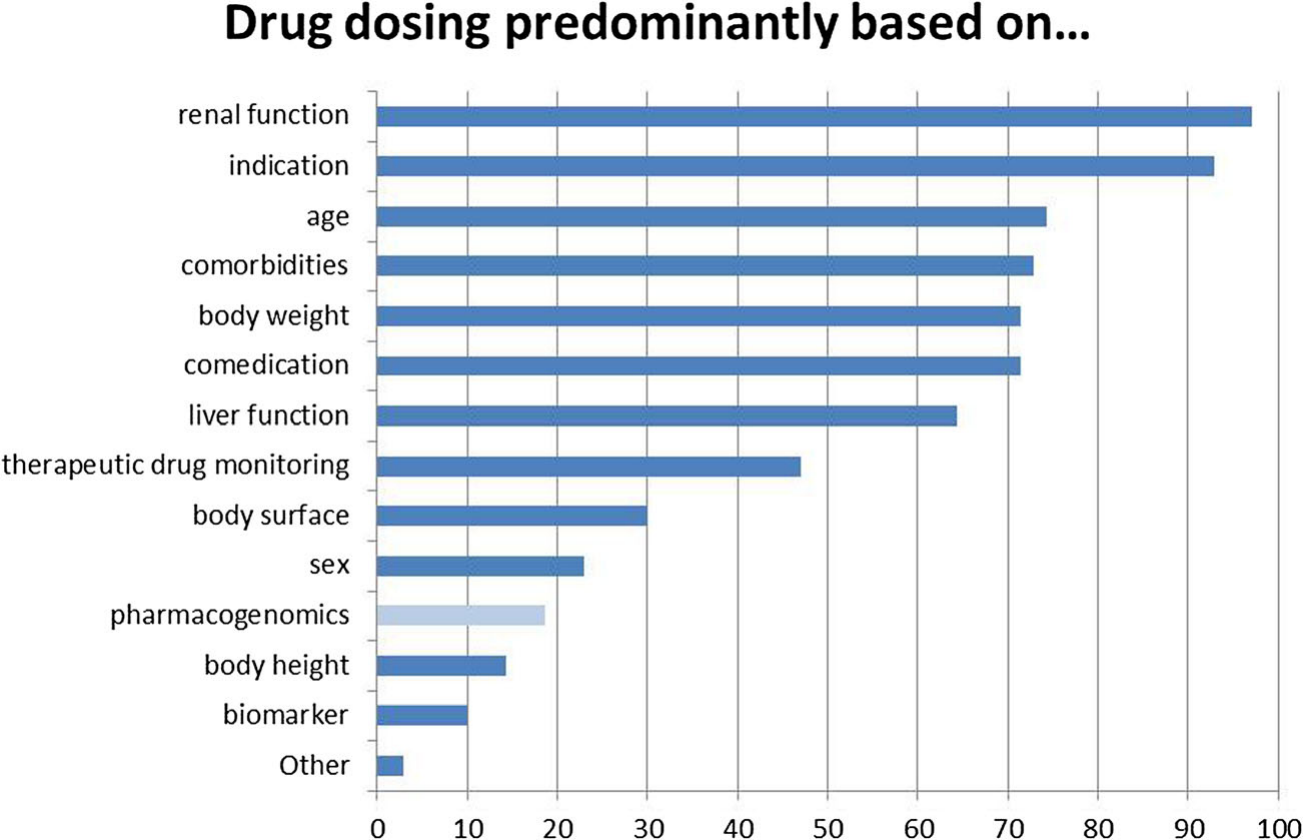
Factors that drug dosing is predominantly based. Given in percentages. Multiple answers possible

The majority of participants agreed that PGx was important in their current practice (84.3%, *agree totally and agree slightly; missing 15.7% disagree totally and disagree slightly*), but 65.7% had not ordered nor recommended a PGx test in the past year. In general, drug dosing was based on multiple factors with pharmacogenomics mentioned as one of the factors taken into account in 18.6% (Fig. 1). The main answers for the reasons why they were not using PGx tests in daily practice were “not applicable” and “not enough knowledge about PGx testing” (each 40.0%, multiple answers possible), followed by lack of reimbursement/insurance coverage (22.9%), and uncertainty about the value of testing (17.1%). Of those that had been ordering a test during the past year (34.3% of the total), a large majority felt that the test results have been useful (91.7%). For potential reasons why pharmacogenomics tests would be ordered, the most often given answer was “not applicable” (41.4%), followed by “for research purposes” and “for preventing or explaining side effects” (25.7% each), and dose adjustments (22.9%).

### Knowledge and educational experience

**Fig. 2:**
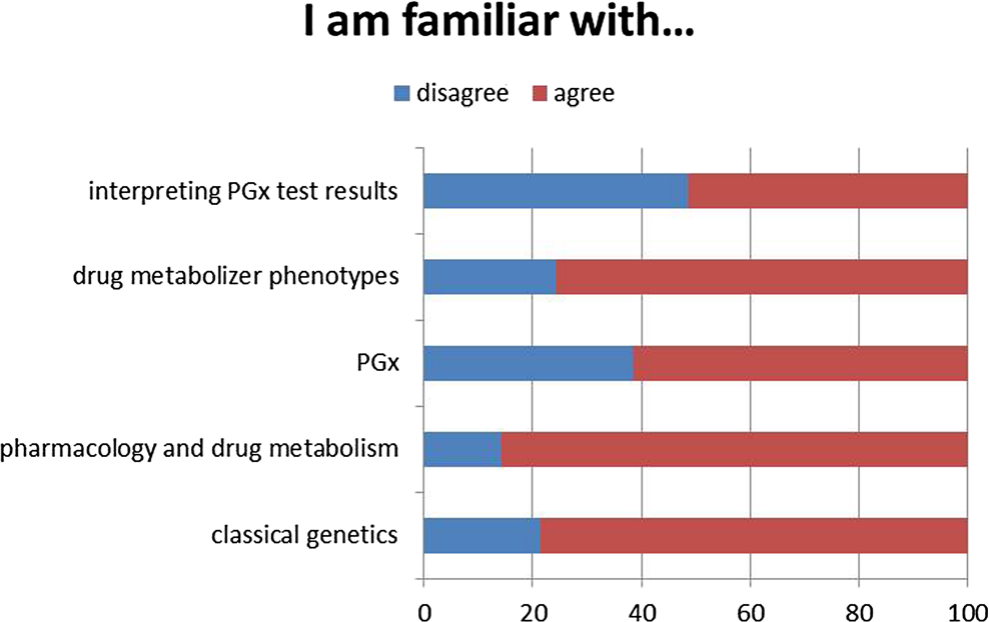
Self-perceived knowledge of participants to our survey. Given in percentages

In majority, participants felt familiar with classical genetics, pharmacology and drug metabolism, and the role of drug metaboliser phenotypes (78.6, 85.8 and 75.7%, respectively, agree totally and agree slightly). In PGx and interpreting PGx test results, participants felt slightly less familiar (61.5 and 51.4%, Fig. 2).

**Fig. 3:**
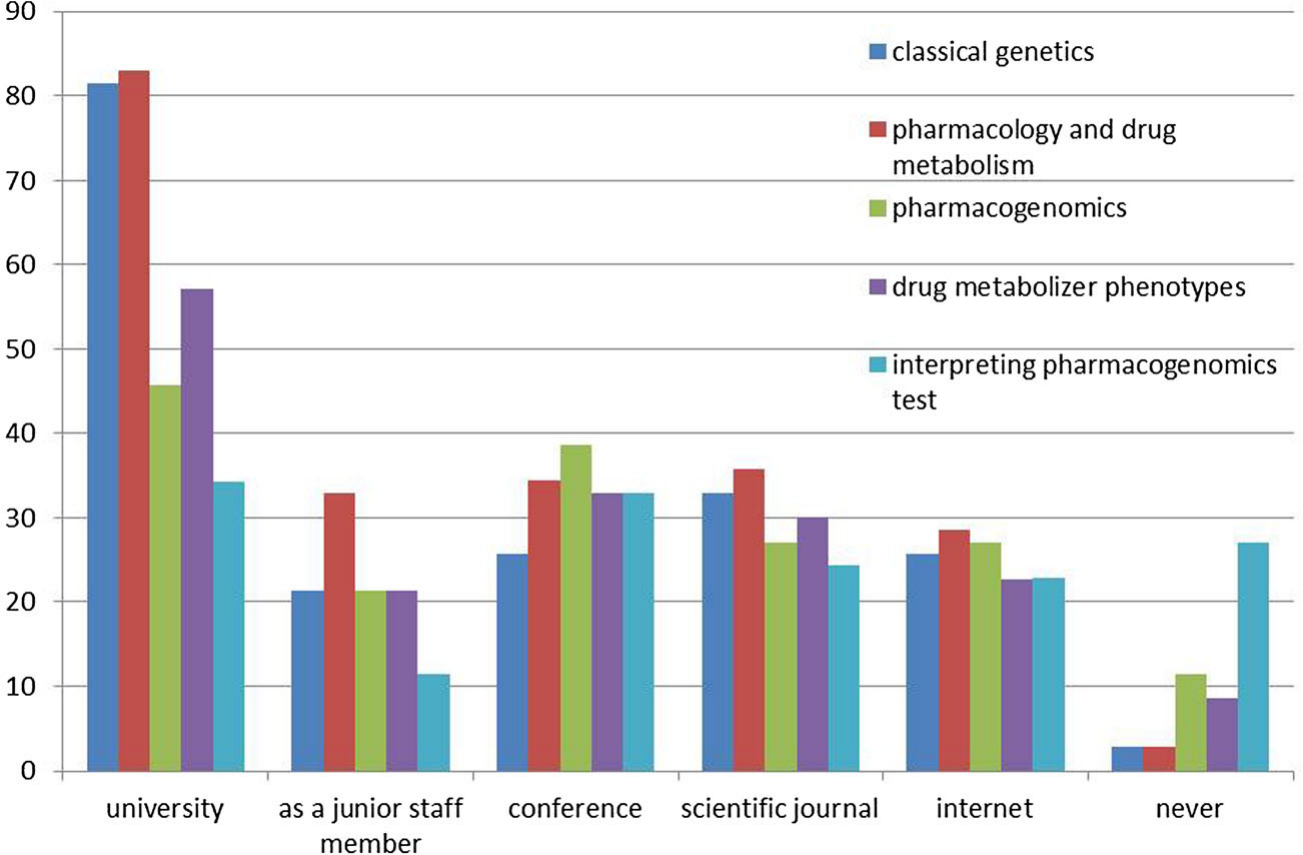
Resources for learning medical topics. Given in percentages. For each topic, multiple answers possible

Nearly on all topics, university was the most important resource for learning (with mainly >50% confirming the importance, multiple answers possible). Resources of postgraduate education, like residency/junior staff membership, conferences, journals or internet were rarely mentioned by more than 30% (Fig. 3).

### Knowledge testing

**Fig. 4:**
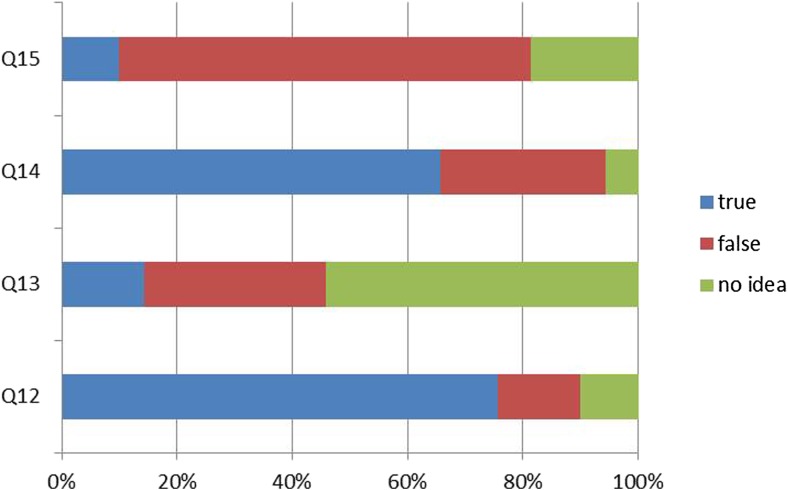
Knowledge testing. Q12: What may be the consequence of a PGx polymorphism? Q13: The EMA currently includes PGx information in the drug labels of how many medications? Q14: What does a PM phenotype indicate? Q15: A person who is a PM for CYP2D6 gets a medication that induces CYP2D6. What may be a consequence?

Performance in knowledge testing in general showed 41% of questions to be answered correctly, 36.6% wrong, and in 22.4% participants indicated “no idea”. According to question, answering varied extensively, with for one question 54.3% answering no idea (Q13), one question with 75.7% correct answer (Q12), and one question with 71.5% incorrect answer (Q15) (Fig. 4).

### Educational needs

Almost half of the participants (41.4%) disagreed to be able to identify drugs that require PGx tests (disagree totally and disagree slightly). And, 37.2% disagreed in being able to use test results accordingly to adjust drug therapy. On the question, “what knowledge would you need to utilize pharmacogenomics for adjustment of therapy?”, 67.1% of participants included better knowledge on drug metabolism, 60.0% better knowledge on the basic concepts of PGx, 55.7% a better evidence that PGx improves clinical outcomes, 52.9% a better knowledge on genetics, 51.4% better knowledge on pharmacology, and more than 40% agreed that they would need support of their working institution (44.3%) and a better ability to apply their knowledge (41.4%).

As preferred learning format on PGx for the future, accredited learning courses (60.0%) and continuing medical education-accredited workshops (55.7%) were picked by over 50% of participants. Furthermore, e-learning courses (47.1%), patient cases and scientific articles (each 44.3%) were mentioned by more than 40% of participants.

## Discussion

With this survey in preparation of the implementation of pharmacogenomics diagnostics in the U-PGx project in Europe, we analysed the specific attitudes, experience and knowledge on PGx in future users [1, 23]. We found a general interest and belief of usefulness of PGx testing in participants. However, the application and interpretation of PGx, when and whom to test, and how to use the results and give therapy recommendations or therapy adjustments, caused uncertainty and needs further improvement. Those results are comparable to results of questionnaires with pharmacists and physicians on pharmacogenomics [8, 9], even though our cohort was slightly more used to application of pharmacogenomics. For example, a survey among Dutch pharmacists revealed 14.7% recent users of PGx diagnostics [27], whereas in our cohort, the percentage was with 34.3% higher. However, the cohort presented consists of future adopters of the PGx implementation study project U-PGx and therefore do not represent the clinical situation on knowledge and attitudes towards PGx in general in Europe.

Implementation sites and their organisational structure to implement PGx varied extensively, thus leading to different sample sizes from each implementation site and country, respectively. Accordingly, this was a heterogeneous group of future adopters of PGx from different medical backgrounds with age and number of work experience ranging extensively. The common background for all participants/study centres was that the use of PGx was already present before the beginning of the study, thereby analysing a collective of future adopters prior to the implementation project. The situation therefore may not be regarded to be representative for the general situation in pharmacogenetic testing within Europe. Respecting the heterogeneity, this survey used a large number of questions on characteristics of participants. Furthermore, this survey was designed to serve as a basis for development of an educational programme within the same European implementation study, addressing the same group of participants and therefore focuses strongly in numbers of questions on education, educational experience, and educational needs.

Results on attitude towards PGx, and interest and usefulness of PGx tests, are probably not generalizable, although in line with results from previous surveys [8, 9, 27]. As depicted above, this cohort was derived from implementation sites within a study trial where some of them are already used to apply PGx in specific medical problems in patient care. Accessory, those results may be partially explained by social desirability bias [28], as participants knew that they would be future users of PGx tests within a study trial.

Also within the knowledge and educational experience section, this social desirability bias may explain the results partially. For example, the majority felt familiar with pharmacology and drug metabolism but on the same side in the educational needs section, wished more education as basis for applying PGx in clinic. A strength of this study is that knowledge was shortly tested, which is not commonly seen in surveys. In general, the grade of knowledge about application and interpretation of pharmacogenomics caused uncertainty and was not at a homogenous level within healthcare professionals working at the clinical sites. This is in concordance with data derived from other European surveys. In a survey on pharmacists and physicians in Greece, over half of the respondents appraise themselves to be unable to explain PGx test results [25] characterising education as a critical step in clinical implementation. Concerning pharmacogenomics education in Universities in South East Europe, a heterogeneous situation was reported and PGx is only rarely delivered as a stand-alone course [24]. In this study, residents and specialist answered with above 80% that they would be unable to interpret test results. And, in a survey among Dutch pharmacists, just 27% felt themselves to be able to interpret test results and to give advises or to treat the patient based on the results [27].

Participants were generally open for education in PGx, which is again in concordance with published data [8, 9, 25]. As specifically the application of PGx in clinic causes uncertainty, one would need educational programmes that focus on clinical implementation. An accreditation of learning would be desired. So far, medical knowledge seems to be mainly generated at universities and the role of postgraduate medical education of pharmacogenomics remains still low. As the importance of education for implementation of PGx into clinics is underlined manifold [5, 8, 9, 11, 17, 23], this might be crucial. A large educational effort has to be done to train for application of this complex topic [10, 12, 29]. Even our specific cohort of participants, who were already opened for PGx and its use in clinic, showed a need for education especially on the actual use of pharmacogenomics, knowing whom and when to test, interpreting test results and giving therapy recommendations.

As pointed out, there are known challenges in conducting trials in pharmacogenomics [2, 7]. This subject might be even more complicated in European countries, not only owing the occurrence of pharmacogenomics variance [29] and the lack of educational programmes in Europe [24] but also due to the heterogeneity of health care systems and number of spoken languages. Especially in the field of pharmacogenomics lays the chance to reduce adverse drug reaction and optimise drug therapy [30, 31]. But, prospective randomised controlled clinical trials are needed using multi-centre international collaborations [32]. A retrospective study including 1017 randomised controlled trials found that 25% of initiated trials remained discontinued. The main reason was patient recruitment with administrative reasons among the most common [33]. European multicentre studies might have an even more increased problem with administrative reasons due to the diversity in political and health systems.

There is a need for European studies, especially in the field of implementing pharmacogenomics into clinic. Realising studies as well as implementation with a European standard remains challenging, and pitfalls such as the diversity of health care systems and educational needs should be attended. However, this challenge has to be taken to exist in the competitive field of research.

## Electronic supplementary material

ESM 1 (PDF 578 kb)
