# Supplementary material for: Medical education in pharmacogenomics—results from a survey on pharmacogenetic knowledge in healthcare professionals within the European pharmacogenomics clinical implementation project Ubiquitous Pharmacogenomics (U-PGx)
Source: Eur J Clin Pharmacol. 2017 Jul 2;73(10):1247–52. doi: 10.1007/s00228-017-2292-5 (PMC5599468; doi:10.1007/s00228-017-2292-5)
Supplement: Supplementary file 1 — (PDF 578 kb) [file 228_2017_2292_MOESM1_ESM.pdf]

U-PGx Clinician`s Questionnaire

QUESTIONNAIRE for PHARMACISTS and PHYSICIANS

To whom the questionnaire addresses:

- physicians (treating and dosing patients)
- hospital pharmacists (therapy adjustments, dose adjustments)

Introduction

This is an anonymous questionnaire to evaluate the views and knowledge of current drug prescribers on pharmacogenomics and its role in individualizing patient treatment. This study is part of the work being carried out by a pan-European research collaboration called Ubiquitous Pharmacogenomics (U-PGx: http://upgx.eu/). The results of this questionnaire will support the development of a short e-Learning Tool on pharmacogenomics for clinicians and pharmacists. This questionnaire is anonymous. However, the outcome of the questionnaire will be published in a scientific journal. We are grateful to you for taking the time to answer the following questions.

This project has received funding from the European Union’s Horizon 2020 research and innovation program under grant agreement No 668353

SECTION 1: EXPERIENCE AND ATTITUDE

1)

Please rank.

	disagree 1	2	3	agree 4
Pharmacogenomics is relevant to my current practice.	◉	○	○	○

2)

How are you involved in the U-PGx project?

☐ I am not involved at all
☐ I treat patients that get tests
☐ I adjust doses/provide recommendations
☐ I order tests by myself
(if other, please specify) ☐ other: ___________________

3)

In general, on which of the following do you predominantly base your drug dosing?
(please select all that apply)

☐ indication
☐ body weight
☐ body height
☐ body surface
☐ renal function
☐ liver function
☐ age
☐ sex
☐ therapeutic drug monitoring
☐ pharmacogenomics
☐ biomarker
☐ comorbidities
☐ comedication
(if other, please specify) ☐ other: ___________________

This project has received funding from the European Union's Horizon 2020 research and innovation program under grant agreement No 668353

4a)

How many times did you order/ recommend a pharmacogenomics test last year?

- ◯ 0
- ◯ 1/ month
- ◯ 2-5/ month
- ◯ 6-10/ month
- ◯ 11-20/ month
- ◯ >20/ month

4b)

Please select why you would not order a test:
(please select all that apply)

- ☐ not applicable
- ☐ concerns about privacy
- ☐ little-or-no, or uncertain about the value of testing
- ☐ lack of insurance coverage for testing
- ☐ not enough personal knowledge about genomic testing
- ☐ not familiar with the legal issues and regulations of pharmacogenomics testing
- ☐ patients' resistance to genomic testing
- ☐ I do not prescribe drugs with genomic tests available or recommended
- (if other, please specify) ☐ other: ____________________

4c)

If ≥1, could you use the test result?

- ☐ Yes
- ☐ No, I did not know how to interpret it.
- ☐ No, because it took too long for the pharmacogenomics test result to come back
- ☐ There was no abnormality.
- (if other, please specify) ☐ other: ____________________

4d)

If ≥1, who paid for the test? (one)

- ◯ patient
- ◯ clinic
- ◯ health insurance
- (if other, please specify) ◯ other: ____________________

5)

Why do you usually order pharmacogenomics tests?
(please select all that apply)

- ☐ obligatory companion diagnostic
- ☐ preventing/ explaining side effects
- ☐ dose adjustments
- ☐ for research purposes
- ☐ tumour diagnostic
- ☐ not applicable
- (if other, please specify) ☐ other: __________________

This project has received funding from the European Union's Horizon 2020 research and innovation program under grant agreement No 668353

SECTION 2: KNOWLEDGE

Please rank your perceived knowledge on a scale.

6a)

	disagree 1	2	3	agree 4
I am familiar with classical genetics.	○	○	○	○

6b)

Where did you learn about this topic?

- ☐ university
- ☐ as a junior staff member
- ☐ conference
- ☐ journal
- ☐ internet
- ☐ never
- (if other, please specify) ☐ other: ________________

6c)

When did you learn about this topic the last time?
(How many years ago?)

- ○ 1-2
- ○ 2-3
- ○ 3-5
- ○ 5-10
- ○ >10
- ○ never

Please rank your perceived knowledge on a scale.

7a)

	disagree 1	2	3	agree 4
I am familiar with pharmacology and drug metabolism.	○	○	○	○

7b)

Where did you learn about this topic?

- ☐ university
- ☐ as a junior staff member
- ☐ conference
- ☐ journal
- ☐ internet
- ☐ never
- (if other, please specify) ☐ other: ________________

7c)

When did you learn about this topic the last time?
(How many years ago?)

- ○ 1-2
- ○ 2-3
- ○ 3-5
- ○ 5-10
- ○ >10
- ○ never

This project has received funding from the European Union’s Horizon 2020 research and innovation program under grant agreement No 668353

Please rank your perceived knowledge on a scale.

8a)

	disagree 1	2	3	agree 4
I am familiar with pharmacogenomics.	○	○	○	○

8b)

Where did you learn about this topic?

- ☐ university
- ☐ as a junior staff member
- ☐ conference
- ☐ journal
- ☐ internet
- ☐ never
- (if other, please specify) ☐ other: ________________

8c)

When did you learn about this topic the last time? (How many years ago?)

- ○ 1-2
- ○ 2-3
- ○ 3-5
- ○ 5-10
- ○ >10
- ○ never

Please rank your perceived knowledge on a scale.

9a)

	disagree 1	2	3	agree 4
I am familiar with the role of drug metabolizer phenotypes (e.g. a poor metabolizer).	○	○	○	○

9b)

Where did you learn about this topic?

- ☐ university
- ☐ as a junior staff member
- ☐ conference
- ☐ journal
- ☐ internet
- ☐ never
- (if other, please specify) ☐ other: ________________

9c)

When did you learn about this topic the last time? (How many years ago?)

- ○ 1-2
- ○ 2-3
- ○ 3-5
- ○ 5-10
- ○ >10
- ○ never

This project has received funding from the European Union’s Horizon 2020 research and innovation program under grant agreement No 668353

Please rank your perceived knowledge on a scale.

10a)

	disagree 1	2	3	agree 4
I am familiar with interpreting the results of pharmacogenomics tests.	○	○	○	○

10b)

Where did you learn about this topic?

- ☐ university
- ☐ as a junior staff member
- ☐ conference
- ☐ journal
- ☐ internet
- ☐ never
- (if other, please specify) ☐ other: ________________

10c)

When did you learn about this topic the last time?
(How many years ago?)

- ○ 1-2
- ○ 2-3
- ○ 3-5
- ○ 5-10
- ○ >10
- ○ never

11)

What sources of information have you used to learn about pharmacogenomics testing and its application to drug prescribing?
(please select all that apply)

- ☐ drug labels (package insert)
- ☐ genetic testing laboratory
- ☐ colleague/ other physician
- ☐ pharmgkb website
- ☐ youtube
- ☐ other internet site
- ☐ journal
- ☐ none
- (if other, please specify) ☐ other: ___________________

This project has received funding from the European Union's Horizon 2020 research and innovation program under grant agreement No 668353

SECTION 3: KNOWLEDGE TESTING

Please answer the following questions to the best of your knowledge (select one answer only per question).

12)

What may be the consequence of a pharmacogenomic polymorphism?

- ○ an individual cannot metabolize any drugs
- ○ an individual has a higher risk for toxicity when using prescription drugs
- ○ a single drug dose is appropriate for a given indication
- ○ individualized dose adjustments should be made according to body surface area

13)

The European Medicine Agency currently includes pharmacogenomics information in the drug labels of how many medications?

- ○ 5-10
- ○ 11-50
- ○ 51-100
- ○ >100

14)

What does a poor metabolizer (PM) phenotype indicate?

- ○ lower drug safety because of poor metabolism
- ○ good drug efficacy because of poor metabolism
- ○ decreased enzyme activity
- ○ increased enzyme activity

15)

A person who is a PM for CYP2D6 gets a medication that induces CYP2D6. What may be a consequence?

- ○ decreased CYP2D6 activity
- ○ no activity of CYP2D6, no consequence
- ○ increased CYP2D6 activity
- ○ the person becomes and intermediate metaboliser (IM) for CYP2D6

This project has received funding from the European Union's Horizon 2020 research and innovation program under grant agreement No 668353

SECTION 4: NEEDS ASSESSMENT

Please rank.

16)

I can identify drugs that require pharmacogenomics testing.	disagree 1	2	3	agree 4
	○	○	○	○

Please rank.

17)

I am confident that I can use the results of pharmacogenomics tests to make an appropriate adjustment to a patient's drug therapy.	disagree 1	2	3	agree 4
	○	○	○	○

18)

To adjust therapy based on pharmacogenomics tests (more often) I would need…

(please select all that apply)

- ☐ better knowledge on genetics
- ☐ better knowledge on pharmacology
- ☐ better knowledge on drug metabolism
- ☐ better knowledge on the basic concepts of pharmacogenomics
- ☐ a better evidence base that pharmacogenomics improves clinical outcomes
- ☐ better ability to apply my knowledge
- ☐ better knowledge of legal regulations
- ☐ support of my working institution
- ☐ insurance coverage
- ☐ expert counsel
- (if other, please specify) ☐ other: ___________________

19)

What would be your preferred format for learning more about pharmacogenomics in the future?

(please select all that apply)

- ☐ scientific article
- ☐ conference talk
- ☐ accredited learning course
- ☐ continuing medical education-accredited workshop
- ☐ patient cases
- ☐ medical app
- ☐ e-learning course
- (if other, please specify) ☐ other: ___________________

20)

How much time would you spend on an e-learning program on pharmacogenomics?

- ○ < 30 minutes
- ○ 30 minutes
- ○ 60 minutes
- ○ 90 minutes

This project has received funding from the European Union's Horizon 2020 research and innovation program under grant agreement No 668353

SECTION 5: DEMOGRAPHICS

Please tell us:

21)
Your year of birth? ______

22)
Your gender? ○ male ○ female

23)
Your profession?
(please select what applies best)
(if other, please specify)

- ○ pharmacist
- ○ physician
- ○ other: ___________________

24)
In which country do you work?

- ○ Austria
- ○ Great Britain
- ○ Greece
- ○ Italy
- ○ Netherlands
- ○ Slovenia
- ○ Spain

25)
Number of years working within your profession since graduating from university?

- ○ 1
- ○ 2-5
- ○ 6-10
- ○ 11-20
- ○ >20

26)
What is your primary practice setting?
(please select what applies best)
(if other, please specify)

- ○ hospital outpatients or ambulatory care
- ○ hospital inpatients
- ○ community-based nursing home
- ○ academia
- ○ research
- ○ other: ___________________

This project has received funding from the European Union's Horizon 2020 research and innovation program under grant agreement No 668353

27)

What is your speciality? (one)

- ○ anaesthetics
- ○ biochemistry
- ○ cardiology
- ○ clinical pharmacology
- ○ emergency or acute medicine
- ○ endocrinology and diabetes
- ○ gastroenterology
- ○ general pharmacy
- ○ genetics
- ○ infectious diseases and microbiology
- ○ haematology
- ○ hospital pharmacy
- ○ nephrology
- ○ neurology
- ○ obstetrics/gynaecology
- ○ oncology
- ○ pathology
- ○ paediatrics
- ○ primary care/ family medicine
- ○ psychiatry
- ○ radiology
- ○ respiratory
- ○ rheumatology
- ○ surgery
- (if other, please specify) ○ other: ___________________

28)

How would you describe your practice setting? (one)

- ○ university/academic
- ○ urban
- ○ suburban
- ○ rural
- (if other, please specify) ○ other: ___________________

29)

Please list any additional comments or feedback here: (max. 250 words)

This project has received funding from the European Union's Horizon 2020 research and innovation program under grant agreement No 668353
